# Study on the Sexuality of Tunisian Women with Systemic Lupus Erythematosus

**DOI:** 10.1192/j.eurpsy.2025.2293

**Published:** 2025-08-26

**Authors:** N. Mseddi, R. Ben Jmaa, F. Frikha, I. Châari, F. Charfeddine, L. Aribi, J. Aloulou

**Affiliations:** 1 Psychiatry B; 2 Internal Medicine, Hédi Chaker Hospital, Sfax, Tunisia

## Abstract

**Introduction:**

Systemic Lupus Erythematosus (SLE) is a systemic disease that can significantly impact women’s lives. Female sexual function is one of the underestimated areas, and few studies have focused on sexual dysfunctions in women with SLE.

**Objectives:**

To study the sexuality of patients with SLE, the factors associated with it, and the relationship with the disease activity stage.

**Methods:**

A descriptive and analytical cross-sectional study was conducted with 38 patients with SLE followed in the internal medicine department at Hedi Chaker University Hospital, Sfax, over a period of 3 months from September to November 2023. We used:

A socio-demographic and clinical data sheet, and the SLEDAI scale for assessing disease activity criteria.

The Female Sexual Function Index (FSFI) scale to study sexuality over the past 4 weeks.

**Results:**

The average age of the patients was 46.63 ± 10.21 years. Among them, 36 were married, and 44.7% had medical-surgical history.

The average age at SLE diagnosis was 37.11, with an average disease duration of 8.95 years. The mean SLEDAI score was 3.63. Absent to moderate activity was present in 92.1% of cases, and high activity in 7.9%.

Photosensitivity was noted in 36.8%, joint involvement in 63.2%, and anemia in 52.6%. Among the patients, 86.8% were on corticosteroids and 26.3% on immunosuppressants.

The average age of onset for sexual difficulties (SD) was 37.28 years, with an average delay of 3.05 years from disease onset.

According to FSFI: the mean score was 58.38 ± 23.412 [2 - 93]. A decrease in overall sexuality was noted in 44.7%, desire in 63.2%, satisfaction in 57.9%, arousal in 52.6%, and lubrication in 39.5%.

The factors correlated with different FSFI domains were:Desire: associated with medical-surgical history (P=0.001), photosensitivity (P=0.028), and anemia (P=0.003). Sexual satisfaction: associated with medical-surgical history (P=0.011), joint involvement (P=0.034). Arousal: associated with medical-surgical history (P=0.01) and anemia (P=0.004).

There were no statistically significant correlations between the disease activity stage and the different FSFI domains. However, the onset of SD was shortened if use of corticosteroid (P=0.005) or immunosuppressive treatment (P=0.038).

**Conclusions:**

Our results indicate that SLE can impair sexuality in various domains, especially if associated pathologies are present. Therefore, special attention should be given to the sexual function of patients with lupus.

**Disclosure of Interest:**

None Declared

